# Temperature Effects and Compensation-Control Methods

**DOI:** 10.3390/s91008349

**Published:** 2009-10-21

**Authors:** Dunzhu Xia, Shuling Chen, Shourong Wang, Hongsheng Li

**Affiliations:** Key laboratory of Micro-inertial Instrument and Advanced Navigation Technology, Ministry of Education, Southeast University, Nanjing, Jiangsu Province, 210096, China; E-Mail: chenshuling318@126.com (S.C.)

**Keywords:** microgyroscope, temperature characteristic, BP neural networks, polynomial fitting, temperature compensation and control

## Abstract

In the analysis of the effects of temperature on the performance of microgyroscopes, it is found that the resonant frequency of the microgyroscope decreases linearly as the temperature increases, and the quality factor changes drastically at low temperatures. Moreover, the zero bias changes greatly with temperature variations. To reduce the temperature effects on the microgyroscope, temperature compensation-control methods are proposed. In the first place, a BP (Back Propagation) neural network and polynomial fitting are utilized for building the temperature model of the microgyroscope. Considering the simplicity and real-time requirements, piecewise polynomial fitting is applied in the temperature compensation system. Then, an integral-separated PID (Proportion Integration Differentiation) control algorithm is adopted in the temperature control system, which can stabilize the temperature inside the microgyrocope in pursuing its optimal performance. Experimental results reveal that the combination of microgyroscope temperature compensation and control methods is both realizable and effective in a miniaturized microgyroscope prototype.

## 1. Introduction

In recent years, the silicon microgyroscope has been used as a kind of inertial device for measuring the angular velocity of an object's motion [1–3]. As is known, it has the merits of small volume, light weight, high reliability and low cost, and it is easy for digitization and intellectualization and suitable for mass production. However the precision and stability of microgyroscopes are prone to be affected by its material of construction, manufacturing technology and other factors such as the temperature of the ambient environment, which could cause the serious drawbacks of low precision and even errors.

Owing to the expansion and centralization in material dimension over temperature, the stiffness of the silicon microgyroscope will change with temperature variation. According to the kinetic equation of the gyroscope, the resonant frequency of a microgyroscope is relevant to the sensitivity and stability as well as its dynamic characteristics, therefore temperature variation has significant impacts on both the output sensitivity, the stability and the dynamic characteristics of microgyroscopes, potentially resulting in a great temperature drift of the entire system.

In [4], the temperature error mechanism of gyroscope caused by Brownian noise was analyzed in detail; the Brownian noise is engendered by gas molecule collisions and the viscous elastic effects of the supporting structure. In [5,6], the temperature error mechanisms of the tuning type and the angular vibrating types of microgyroscopes were analyzed in terms of systematic Brownian noise, however Brownian noise mainly determines the resolution ratio of the gyroscope but has little impact on the zero bias error. In [7], systematic identification was accomplished through some testing methods, and the mechanism of gyroscope temperature error was mainly analyzed in terms of the structural resonance frequency variation caused by temperature changes. In [8], the temperature model of a silicon gyroscope was deduced from the point of view of the Seebeck effects of the silicon material, but only the temperature error caused by Young's modulus changes over the temperature variation were discussed. In [9], the modeling of detection capacitance analysis for a tuning fork vibratory microgyroscope fabricated by bulk silicon micromachining was presented, and the deviations of the frequencies and dynamic characteristics were accurately calculated, which provides a reference for the design of a temperature compensation method and a robust structural design for microgyroscopes. In [10], the temperature dependent drift and the noise characteristics of the packaged silicon MEMS gyroscopes are thoroughly investigated. The resonant frequency, quality factor, AGC voltage and signal drifts were all subjected to various temperature environments from −60 °C and +60 °C. However, no schemes for effective temperature compensation were suggested.

Above all, the performance of a microgyroscope is greatly affected by the power consumption and ambient temperature variation, and the effect of environmental temperature becomes one of the most important sources of error in microgyroscopes. Therefore some effective measures should be taken to reduce the influence of temperature, including temperature compensation and temperature control, which should be both efficient and feasible for improving the precision and stability of the microgyroscope.

Two different methods are proposed in this paper. The first one is a temperature compensation method, the other one is the temperature control method. For the temperature compensation method, a temperature drift model of the microgyroscope is constructed first. It is then used to estimate the current zero bias which is then subtracted from the actual output to obtain the compensated output of the microgyroscope. As for the temperature control method, a microprocessor is employed to stabilize the temperature inside the gyroscope casing.

## 2. Temperature Effects on the Performance of Microgyroscopes

**Figure 1.**
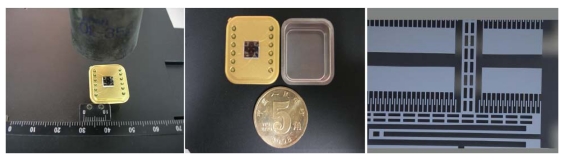
The package and SEM photos of a microgyrosocpe.

In this paper, the symmetrical and decoupled microgyroscope used is essentially a two linear vibratory gyroscope to detect rotation angular rate with the oscillating components. Its simple model can be described as a total four DOFS system including proof mass, drive component, sense component, and damping elements. Figure 1 shows the package and SEM photos of the structure.

The Coriolis dynamic equations of Z-axis microgyroscope is:

(1) $$m_{x}\overset{••}{x}+D_{x}\overset{•}{x}+K_{x}x=F_{e}+F_{dx}+m_{x}\omega_{z}^{2}x-2m_{x}\omega_{z}\overset{•}{y}+m_{x}\overset{•}{\omega_{z}}y$$

(2) $$m_{y}\overset{••}{y}+D_{y}\overset{•}{y}+K_{y}y=F_{dy}+m_{y}\omega_{z}^{2}x+2m_{y}\omega_{z}\overset{•}{x}-m_{y}\overset{•}{\omega_{z}}y$$

where *m_x_* and *m_y_* denote the mass of the driving and sensing segments of the microgyroscope, respectively. *D_x_* denotes the damping coefficient of the flexible shaft connecting the substrate and the drive frame, *D_y_* denotes the damping coefficient of the flexible shaft connecting the substrate and the sense frame. *K_x_* is the spring coefficient of the flexible shaft connecting the substrate and the drive frame, *K_y_* is the spring coefficient of the flexible shaft connecting the substrate and the sense frame. *ω_z_* is the angular rate around the Z-axis. *F_e_* = *F_d_* sinω*_d_t* and *F_d_* represents the amplitude of the electrostatic driving force, ω*_d_* indicates its angular frequency.

According to the kinetic equations, ignoring the crosstalk interference terms, Equations (1) and (2) can be simplified to the following kinetic equations:

(3) $$\overset{••}{x}+2\varsigma_{x}\omega_{x}\overset{•}{x}+\omega_{x}^{2}x=\frac{F_{e}}{m_{x}}$$

(4) $$\overset{••}{y}+2\varsigma_{y}\omega_{y}\overset{•}{y}+\omega_{y}^{2}y=2\omega_{z}\overset{\text{From the above analysis}}{x}$$

where *ς_x_* = *D_x_/*2*m_x_ω_x_* and *ς_y_* = *D_y_/*2*m_y_ω_y_* denote the damping ratios of drive axis and the sense axis of the microgyroscope, respectively.

Various factors can impact the performance of microgyroscope, including the changes of the characteristics of its material, and variations of the electrical characteristics of its peripheral circuit. Noticeably, temperature has a remarkable influence on various aspects, including the resonant frequency, the quality factor and the zero bias.

### 2.1. Temperature Effects on the Resonant Frequency of a Silicon Gyroscope

Considering that the substrate of the microgyroscope is glass, and its supporting beam is made of monocrystalline silicon, the Young's modulus changes with the temperature variation, so their relationship can be expressed as:

(5) $$E=K_{E}TE_{0}$$

where *K_E_* ≈ −5 × 10^−5^ *N*/*MT* denotes the temperature coefficient of the silicon material, *E*_0_ is the temperature coefficient of monocrystalline silicon at Kelvin temperature.

Meanwhile, a strain exists between the supporting beam and the substrate owing to the difference in thermal dilation coefficient among different materials, thus the strain quantum can be expressed as:

(6) $${ɛ}_{th}=\int_{T_{0}}^{T}(\alpha_{s}-\alpha_{g})dT=(\alpha_{s}-\alpha_{g})\Delta T$$

where *α_s_, α_g_* are the thermal expansion coefficients of monocrystalline silicon and glass respectively, Δ*T* is the variable temperature.

Therefore the variable quantity of residual stress is:

(7) $$\Delta \sigma =(\frac{E_{0}}{1+v}){ɛ}_{th}=\frac{(\alpha_{s}-\alpha_{g})E_{0}\Delta T}{1+v}$$

where ν denotes the Poisson ratio of the monocrystalline silicon.

The relationship between the natural frequency and the Young's modulus, as well as the residual stress of the supporting beam, can be expressed as [11]:

(8) $$\omega =\sqrt{\frac{4EhW^{3}+\pi {AL_{1}}^{2}\sigma /4}{{mL_{1}}^{3}}}$$

where *E* denotes the Young's modulus of the silicon material, σ denotes the residual stress, *h* and *L*_1_ denote the height and the length of the structure respectively, *m* denotes its mass, *A* denotes the area of structure, and *W* denotes the stability index.

From the above analysis, the Young's modulus *E* and the residual stress σ are proportional to the temperature variation. Moreover it can be seen in Equation (8) that the resonant frequency is positively correlated with *E* and σ. Therefore it can be deduced that the resonant frequency of a microgyroscope has a proportional relation with temperature changes.

### 2.2. Temperature Effects on the Quality Factor of a Silicon Gyroscope

**Figure 2.**
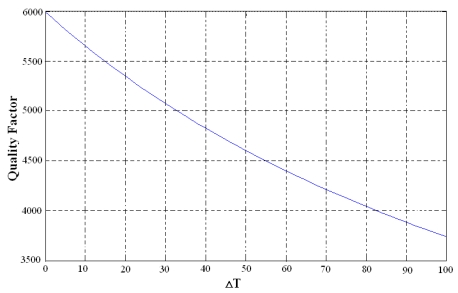
Simulation of the relationship between Q and temperature.

According to [12], it is supposed that only a rarefied gas will provide the main damping, thus the quality factor of the system is:

(9) $$Q=\frac{M_{P}\omega_{0}}{C_{r}}$$

where *M_p_* = *Ahρ* is the mass of the electrode plate, *A, h, ρ* denotes the area, thickness and density of the electrode plate respectively. *C_γ_* = 4*√(*2*/π)√(M/RT)*·*PA* is the damping coefficient of the rarefied gas in the free gas molecule mode. *M* is the gas mol mass. *R* = 8.31 kg·m^2^/sec^2^/K is the generalized mol constant of gas, *P* is the gas pressure. In this vacuum packaged microgyroscope, given that the volume of gas is fixed, according to Charles's rule: *P*/*T* = *Constant*, the quality factor of the system is:

(10) $$Q=\frac{h\rho \omega_{0}}{4}\sqrt{\frac{\pi }{2}}\sqrt{\frac{R}{MT}}C$$

where *C* is a given constant, the relationship between quality factor *Q* and temperature can be seen directly through the Matlab simulation shown in Figure 2.

### 2.3. Temperature Effects on the Sensing Output of a Silicon Gyroscope

The vibrating displacement in drive mode of the microgyroscope is:

(11) $$\begin{array}{l} x(t)=\frac{F_{d}/m_{x}}{\sqrt{{\left({\omega_{x}}^{2}-{\omega_{d}}^{2}\right)}^{2}+4{\varsigma_{x}}^{2}{\omega_{x}}^{2}{\omega_{d}}^{2}}}\sin(\omega_{d}t+\varphi )\\ =\frac{F_{d}/m_{x}}{\sqrt{{\left({\omega_{x}}^{2}-{\omega_{d}}^{2}\right)}^{2}+4{b_{1}}^{2}{\omega_{d}}^{2}}}\sin(\omega_{d}t+\varphi )\\ =\frac{\left(F_{d}/m_{x})\sin(\omega_{d}t+\varphi \right)}{\sqrt{{\left(\frac{4K_{E}E_{0}hW^{3}T+\overset{\pi AL_{1}^{2}\sigma }{\;}/\underset{4}{\;}}{mL_{1}^{3}}-\omega_{d}^{2}\right)}^{2}+16K_{B}T\omega_{d}^{2}(\frac{0.3502\rho A_{m}}{\pi d_{m}^{3}m\sqrt{\pi m}}+\frac{0.3502\rho A_{1}}{\pi d_{1}^{3}m\sqrt{\pi m}})}} \end{array}$$

**Figure 3.**
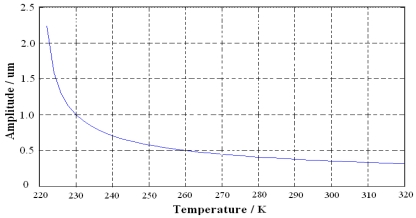
Simulation of relationship between the output amplitude and the temperature.

According to [13], the resonant frequency *ω_x_* and damping coefficient *b*_1_ in drive mode of microgyroscope are proportional to the temperature. Thereby the amplitude of vibrating displacement is negatively correlated to the temperature, that is, the amplitude of vibrating displacement in drive mode decreases when temperature increases, which can be seen from the simulation shown in Figure 3.

Similarly, the vibrating displacement in sense mode is:

(12) $$\begin{array}{l} y(t)=\frac{F_{c}}{\sqrt{{\left({\omega_{y}}^{2}-{\omega_{d}}^{2}\right)}^{2}+4{\varsigma_{y}}^{2}{\omega_{y}}^{2}{\omega_{d}}^{2}}}\sin(\omega_{d}t+\varphi +\gamma )\\ =\frac{F_{c}}{\sqrt{{\left({\omega_{y}}^{2}-{\omega_{d}}^{2}\right)}^{2}+4{b_{2}}^{2}{\omega_{d}}^{2}}}\sin(\omega_{d}t+\varphi +\gamma ) \end{array}$$

where *ω_y_* denotes the resonant frequency and *b*_2_ the damping coefficient. Similar to the simulation in drive mode, the vibrating amplitude in sense mode is negatively correlated to temperature.

## 3. Temperature Characteristics of a Microgyroscope

In order to determine the actual effects of temperature on the performance of a gyroscope, a vacuum encapsulated microgyroscope named B34 was adopted, and relevant experiments designed and carried out to verify the above theoretical analysis.

**Figure 4.**
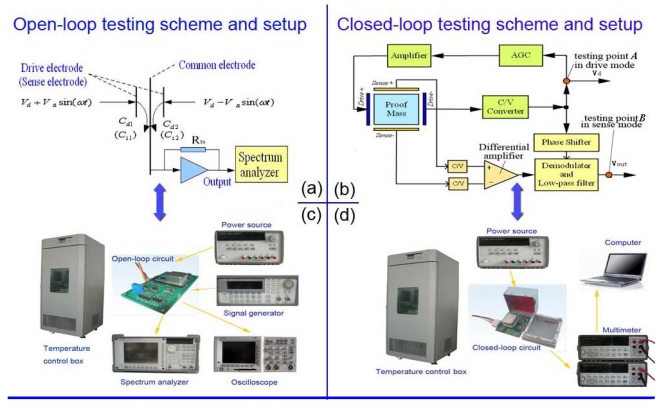
Temperature testing schemes and setup of microgyroscope.

First of all, an open-loop driving circuit was adopted to drive the microgyroscope for testing its resonant frequency and the quality factor under the circumstances of the temperature varying from −40 °C to 60 °C. The waveform generator provides the sinusoidal signal to drive the microgyroscope. Figure 4 shows the setup of the temperature experiment. The microgyroscope in the temperature control box is driven by an open-loop driving circuit. Its output is connected to a spectrum analyzer and oscilloscope, respectively. The oscilloscope exhibits the displacement waveform of the vibrating capacitance while the spectrum analyzer shows its corresponding spectrum output. Figure 4(a) shows the scheme of the open-loop drive circuit testing of the microgyroscope. *C_d1_* = *n_d_ε*_0_*h*(*L* + *x*)/*d*, *C_d2_* = *n_d_ε*_0_*h*(*L* − *x*)/*d*, where *n_d_* denotes the number of drive combs; *ε*_0_ denotes the dielectric constant; *h* is the thickness of comb; *d* denotes the distance between two adjacent combs while *L* is the length of their overlapping part; *x* = *A_x_*sin(*ωt* + *θ*) is the displacement in drive mode. The drive signals *V_d_* ± *V_a_*sin(*ωt*) are symmetrically applied on the drive electrodes to get the testing output *V_td_* across the feedback resistor *R_ts_* at the common electrode.

(13) $$\begin{array}{ll} V_{td} & =(\frac{d[(V_{d}+V_{a}\sin\omega t)C_{d1}]}{dt}+\frac{d[(V_{d}-V_{a}\sin\omega t)C_{d2}]}{dt})R_{ts}\\ & =\frac{2n_{d}{ɛ}_{0}hV_{a}R_{ts}}{d}\sin\omega t+\frac{2n_{d}{ɛ}_{0}hV_{a}R_{ts}x}{d}\omega \cos\omega t\\ & =\frac{2n_{d}{ɛ}_{0}hV_{a}R_{ts}}{d}\sin\omega t+\frac{n_{d}{ɛ}_{0}hV_{a}R_{ts}A_{x}}{d}\omega \sin(2\omega t+\theta )+\frac{n_{d}{ɛ}_{0}hV_{a}R_{ts}A_{x}}{d}\omega \sin\theta \end{array}$$

In Equation (13), the second part of *V_td_*, i.e., the double-times frequency component, is relevant to the vibrating amplitude *A_x_*. By sweeping the frequency *ω* of drive voltage, the frequency response of vibrating amplitude can be recorded, so its peak of the frequency response is just the resonant frequency *ω_d_*, and its quality factor *Q* can be computed by *ω_d_*/*ω*_−_*_3db_* (−3 db declining frequency bandwidth).

During the course of measurement, the microgyroscope and the circuit are statically mounted. The temperature value in the temperature control box rises from −40 °C to 60 °C in 10 °C intervals. The temperature at each sampling point is maintained for sixty minutes before testing to ensure that the temperature in the box is uniformly distributed and the microgyroscope is fully heated. In this way, any difference between the inner and outer temperature of the microgyroscope casing can be effectively reduced. The testing results are provided in Table 1.

**Figure 5.**
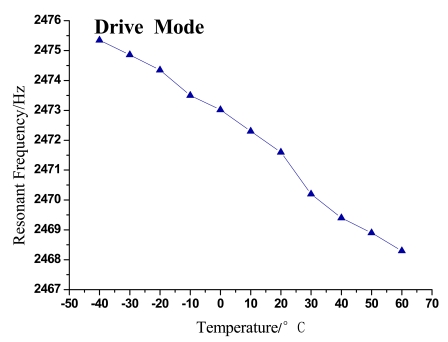
Trend of resonant frequency with temperature in drive mode.

**Figure 6.**
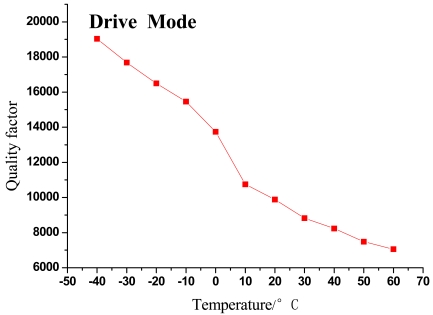
Trend of quality factor change with temperature in drive mode.

**Figure 7.**
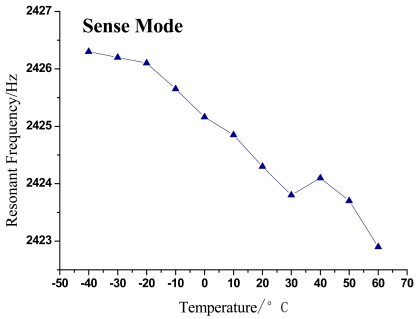
Trend of resonant frequency change with temperature in sense mode.

**Figure 8.**
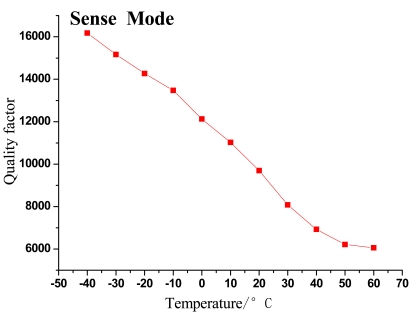
Trend of quality factor change with temperature in sense mode.

The changing trends of gyroscope's resonant frequency and quality factor with temperature variation can be seen in Figures 5–8.

As shown in the above figures, it is obvious that the testing results shown in Figures 5 and 7 validate the analysis in Section 2 and are in good accord with Equation (9), while Figures 6 and 8 are in good accord with Equation (10) and the simulation in Figure 2.

According to the data in Table 1 and Figure 5 and 7, the resonant frequency of the microgyroscope descends linearly while the temperature increases, and its variable magnitude is within 10 Hz from −40 °C to 60 °C. It is noticeable in Figures 6 and 8, that the quality factor changes drastically under the low temperature circumstance, much higher than that of normal temperature. Moreover its changing tendency mitigates when the temperature climbs higher than 30 °C. The reason for this phenomenon is that most of the little residual gas is absorbed under low temperature, and it is released when the temperature increases. However, only a little gas is absorbed in the vacuum packaged gyroscope, therefore the quality factor varies slowly as only a little gas is released when temperature increases.

The second step is to test other performances of the same microgyroscope by the closed-loop driving circuit. According to the prior art [14], the closed-loop circuit can realize the automatic tracking of the resonant frequency drift of the microgyroscope with temperature in drive mode. Figure 4(c) shows a schematic of the closed-loop drive circuit. The variation of the vibrating capacitance in the drive– combs is converted to the corresponding voltage *V_d_* by C/V converter, and then its amplitude can be stabilized via the automatic gain controller (AGC) module. The feedback signal is further amplified to drive the drive+ combs. In sense mode, the capacitance variations of sense+ and sense– combs are simultaneously converted to voltage, and the differential value is demodulated and filtered through the low-pass filter to get the *V_out_*. *V_d_* is changed by the phase shifter and used as reference by the demodulator. The thermal features of its vital parameters such as the zero drift and the output amplitude should be the primary consideration. During this phase, when the microgyroscope is held still, the amplitude of drive circuit [testing point A shown in Figure 4(c)] at normal temperature and varying temperature are tested, respectively, and the zero bias of microgyrosocpe [testing point B shown in Figure 4(c)] is tested over temperature changes.

**Figure 9.**
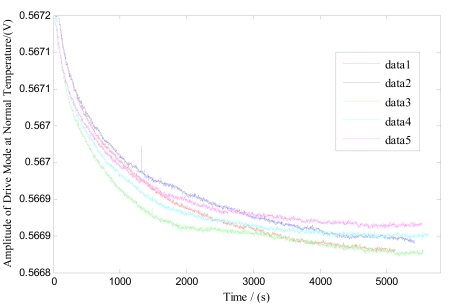
Closed-loop drive circuit test of zero bias of microgyroscope at normal temperature.

Firstly, five groups of zero microgyroscope bias are recorded, respectively, at normal temperature under the same conditions. The test began from the start-up time once the microgyroscope is powered on, and a 30-minute interval is set between each successive group. From the results shown in Figure 9, it is obvious that the drive circuit takes a relatively long time to heat up and reach the thermal equilibrium state. Secondly, the microgyroscope is placed in the temperature control box, and its closed-loop driving amplitude and zero bias are measured, respectively, as the temperature rises from −40 °C to +60 °C in 20 °C steps. The temperature is then kept for one hour at every test point, which can ensure that the microgyroscope is fully heated and the temperature field inside the microgyroscope is homogeneously distributed.

**Figure 11.**
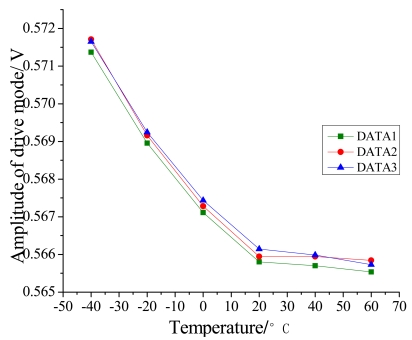
Trend of drive amplitude change with temperature.

**Figure 13.**
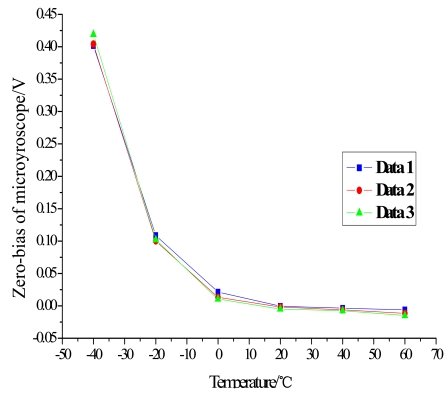
Trend of zero bias change with temperature.

During the temperature experiments, the mirogyroscope is placed still in the temperature control box. At each temperature point it takes one hour to reach thermal balance before the recording of the zero bias of the microgyroscope. Each sampling point is recorded for sixty minutes. Then the average output of the microgyroscope at each temperature point is calculated, so the overall trend of closed-loop drive amplitude and zero bias over temperature changes can be shown as in Figures 11 and 13. In general, the testing results agree well with the analysis of Equations (11) and (12). From the above testing experiments, we can acquire some important knowledge about the thermal characteristics of microgyroscope designed in the laboratory, which could provide useful help for further compensation and temperature control.

## 4. Temperature Compensation Modelling Methods

According to the analysis discussed in Section 2 and the testing results in Section 3, the temperature variation has multiple effects on the performance of a microgyroscope. Consequently the zero drift and the scale factor of gyroscope's output fluctuate with temperature variation, resulting in impairments of the precision and the stability. Therefore, it is necessary to conduct the research on temperature compensation and temperature control for the microgyroscope. In this paper, the BP neural network model and the polynomial fitting for compensation are proposed, then the former method is simulated by Matlab tools, and the latter one is applied in the actual system because of its simplicity and effectiveness. Finally, considering that the temperature compensation could not suppress the zero bias drift completely, an effective temperature control system is adopted to minimize temperature impacts on the gyroscope's performance.

### 4.1. BP Neural Network Modeling Compensation

BP (Back Propagation) neural networks are widely applied in function approximation, pattern recognition, classification and data compression [15–17]. It is a kind of forward neural network with multi layers in the one-way transmission, and generally has the hidden layers and output-layer. BP neural networks have the advantages of nonlinear fitting and mode identification capability, regardless of the mathematical model of the sensors and various nonlinear factors. Its structure is comparatively simpler than other types of artificial neural networks. Moreover it is self-adaptive in constructing a mathematical model after several repetitive learning and testing phases. Thus with the help of BP neural network, it is feasible and effective to obtain a high-precision simulation model without knowing exactly the heat conduction mechanism inside the microgyroscope.

The number of the adopted neurons and the hidden layers mainly depends on the complexity degree of the issue to be solved because BP neural networks require that the transfer function can be differential everywhere. The Sigmoid activation functions (tan-Sigmoid, log-Sigmoid and linear-Sigmoid) are usually adopted as the transfer function, and the linear function is regularly used as the output layer. Furthermore, each node in this structure has close-value function, and the weighted effects of upper layer are transmitted to lower layer through the transfer function.

The essence of nonlinear fitting by BP neural networks is that through self-studying the BP neural network can determine the corresponding relationship between the input and output, which is memorized as a connecting weight value in the network. Therefore the BP neural network structure adopted for temperature modeling and compensation can be selected as follows: first, the original sampled data, i.e., the zero bias over the temperature is normalized. Next, since it aims to build the model between temperature T and zero bias *V*_bias_, each input and output layer is selected with only one neuron. To ensure the precision of the curve fitting, more hidden layers with more neurons are required, however, on the contrary, this will add excessive computing to reach the learning and testing phases. Besides, it may affect the convergence rate and cause unintended fitting errors in case of improper training. There is no exact rule governing the selection of a particular number of hidden layers and neurons, and three hidden layers are chosen through various simulation trials and each layer has ten neurons for trade off. The transfer function is connecting input layer with hidden layers, while that connecting hidden layer and output layer are pure linear Sigmoid function. This structure can provide a satisfactory convergence effects while speeding up the training procedure at the same time.

**Figure 14.**
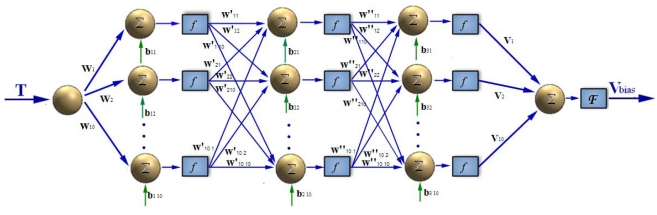
Structure of the BP neural network adopted for modeling.

**Figure 15.**
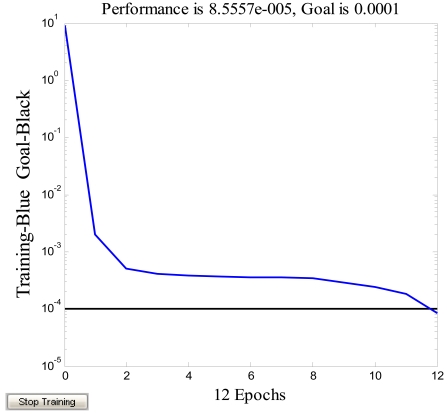
Training of network.

**Figure 16.**
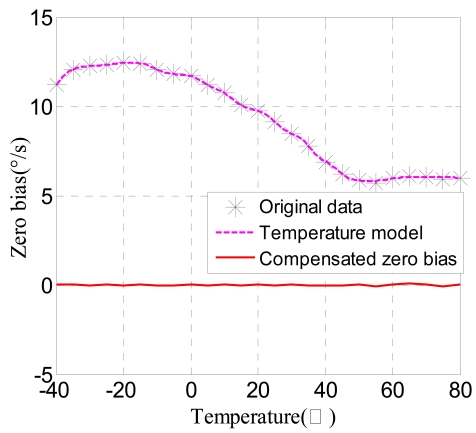
BP model compensation.

The structure is shown in Figure 14, *w_i_* (*i* = 1, 2, …, 10) is the weight value connecting the input layer and the middle layer, *w″_1i_* (*i* = 1, 2, …, 10) is the weight value connecting the first and second hidden layer; *w″_2i_* (*i* = 1, 2, …, 10) is the weight value connecting the second and third hidden layer; *v_j_* (*j* = 1, 2, …, 10) is the weight value connecting the middle layer and the output layer. *b_ij_* (*i* = 1, 2, 3; *j* = 1, 2, …, 10) is the threshold value. *f* is the transfer function tan-Sigmoid, *F* is the transfer function linear-Sigmoid. Secondly, to speed up convergence and reduce the error, the trainlm function is adopted in the training. Taking the temperature as the input and the zero drift of microgyroscope as the output to train this network through the reversal Levenberg-Marquadt algorithm, with the maximum twelve training steps the training target error approaches 0.0001. As shown in Figure 15, the training target error results can basically meet the requirement after only training four times. Finally, all the trained networks weights and offsets are preserved as constant coefficients, thus the BP neural network of temperature model is successfully built up. The final simulation of the model is shown in Figure 16.

After the network model is built, the current temperature is used as the input value to get the corresponding zero bias. Then it is subtracted from the actual output to attain the compensated zero bias of the gyroscope. In the Matlab simulation, the original temperature is substituted to get the compensated zero bias curve, which is obviously near zero and almost becomes a straight line approaching zero in Figure 16.

**Figure 17.**
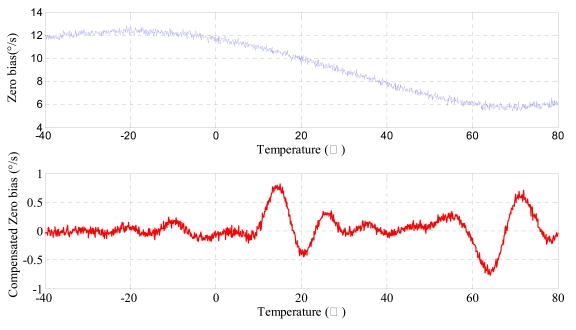
Verification of BP neural networks compensation effects.

In order to verify the effectiveness of the compensating model, the gyroscope is put in the thermal control box, and the ambient temperature is controlled to increase from −40 °C to 80 °C by 10 °C step. At each temperature point the zero bias is recorded for thirty minutes, thus the compensated zero bias curve is attained through the built up line compensating model in the computer. As can be seen in Figure 17, the maximum absolute value of zero bias stability decreases remarkably, from 12.3310°/s to 0.756°/s, after compensation, which will provide a reference for comparison with other compensating methods.

Theoretically the BP neural networks modeling provides excellent results for the temperature compensation. However in the first place, massive calculation is incorporated so that it requires high processing capability of the microprocessor and a large database. Secondly, the network needs re-trained to update parameters once the input samples increase due to its poor generalization. Moreover it is relatively complex to implement real-time processing in current chosen microprocessors. Another way of building temperature model of microgyroscope is based on the numerical analysis of its actual output and corresponding temperature. Polynomial fitting has been widely applied in terms of numerical analysis, and the least mean square curve fitting is liable to get overall optimal results. Therefore the polynomial fitting method is proposed for simplicity and effectiveness, and compared to the BP network method, it can provide similarly satisfying compensating effects.

### 4.2. Polynomial Fitting

The main idea of the polynomial fitting compensation is as follows: the correlation between the temperature and the zero bias of microgyroscope can be found through experiments, and its mathematical expression can be obtained through polynomial fitting as the temperature is changed from −40 °C to 80 °C. The mathematical function is memorized in the microprocessor. Thus the corresponding compensation value for each real time temperature tested is calculated and subtracted in the actual output of gyroscope. Ultimately the final compensated zero bias of the gyroscope is attained.

While constructing the compensation model, special consideration must be paid to the precision, practicability and complexity of the model to satisfy the engineering requirements. With respect to the real-time performance of the compensating system and the control and operation capability of the C8051F360, the least mean squares (LMS) curve fitting is utilized as it is simple and easy for constituting the temperature model of the zero bias of gyroscope.

Using the high-order polynomial to describe the approximate function (regression equation) relationship of the experimental data (*x*_i_, *y*_i_) (where *x*_i_ denotes the input, and *y*_i_ denotes the corresponding output, *i* = 1, 2, …, *n*), the following expression can be obtained:

(14) $${V_{i}=y_{i}-\sum_{j=0}^{m}a_{j}{\text{x}}_{i}^{j}\;}_{i=1,2,\cdots ,n}$$

where *V*_i_ denotes the error between the tested value and the result calculated by the regression equation. According to the LMS theory, the square of *V*_i_ should be set to the minimum to obtain the optimum value of the coefficient *a*_j_:

(15) $$\varphi \left(a_{0},a_{1},\cdots ,a_{m}\right)=\sum_{i=1}^{n}V_{i}^{2}={\sum_{i=1}^{n}\left(y_{i}-\sum_{j=0}^{m}a_{j}{\text{x}}_{i}^{j}\right)}^{2}\to \min$$

From which we can deduce the following canonical Equation(16):

(16) $$\frac{\partial \varphi }{\partial a_{k}}=-2\sum_{i=1}^{n}\left[\left(y_{i}-\sum_{j=0}^{m}a_{j}{\text{x}}_{i}^{j}\right){\text{x}}_{i}^{k}\right]=0$$

Then the linear equation for computing *a*_0_, *a*_1_, *…, a*_m_:

(17) $$\left[\begin{array}{llll} n & \sum_{i=1}^{n}x_{i} & \cdots & \sum_{i=1}^{n}{\text{x}}_{i}^{m}\\ \sum_{i=1}^{n}x_{i} & \sum_{i=1}^{n}x_{i}^{2} & \cdots & \sum_{i=1}^{n}x_{i}^{m+1}\\ \cdots & \cdots & \cdots & \cdots \\ \sum_{i=1}^{n}{\text{x}}_{i}^{m} & \sum_{i=1}^{n}x_{i}^{m+1} & \cdots & \sum_{i=1}^{n}x_{i}^{2m} \end{array}\right]\cdot \left[\begin{array}{l} a_{0}\\ a_{1}\\ \cdots \\ a_{m} \end{array}\right]=\left[\begin{array}{l} \sum_{i=1}^{n}y_{i}\\ \sum_{i=1}^{n}x_{i}y_{i}\\ \cdots \\ \sum_{i=1}^{n}x_{i}^{\text{m}}y_{i} \end{array}\right]$$

The solution for Equation (17) is the optimum value for *a*_j_(*j* = 0, 1, …, *m*).

**Figure 18.**
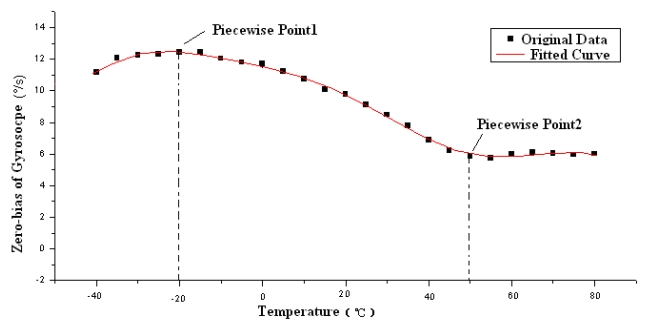
Fitted curve of zero bias of microgyroscope with temperature.

In the temperature experiments, the zero bias output of the gyroscope is recorded while the temperature increases from −40 °C to 80 °C in 5 °C intervals, which can be seen in Table 2 and Figure 18.

After fitting the experimental curve with the LMS theory, we can separate it into three segments to reduce the order of polynomial and minimize the error.

When −40 °C ≤ *T* ≤ −20 °C:

$$V_{\text{bias}}=-10.3-19.2567T-1.139T^{2}-0.02933T^{3}-0.00028T^{4}$$

When −20 °C < *T* ≤ 50 °C:

$$V_{\text{bias}}=-101.6788-0.64109T-0.01231T^{2}-0.000089526T^{3}$$

When 50 °C < *T*≤ 80 °C:

$$V_{\text{bias}}=2370.0786-146.13135T+3.413T^{2}-0.03502T^{3}+0.0001333T^{4}$$

The fitted curve is shown in Figure 18:

## 5. Design of the Temperature Compensation System and Experimental Results

### 5.1. Design of the Temperature Compensation System

**Figure 19.**
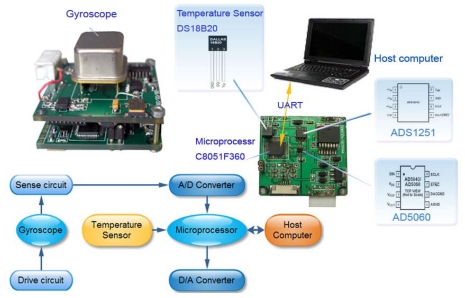
Diagram of the temperature compensation system.

The temperature compensation scheme is shown in Figure 19. First of all, the developed miniaturized analog gyroscope works well. The uncompensated analog signal output of the gyroscope is first converted into a digital value by an ADS1251 AD converter (Texas Instruments Company). Meantime, the temperature value is measured by a DS18B20 digital temperature sensor (Dallas Semiconductor) and transmitted to a C8051F360 microprocessor (Silicon Labs Company) which is the core of whole system. The fitting polynomials are previously stored in the flash memory of the C8051F360 and used to calculate the compensation value in terms of the measured temperature, and then the compensated gyroscope signal is converted into analog signal by an AD5060 (Analog Device Company) and transferred out to become a analog gyroscope again. On the other hand, to realize a digital gyroscope, the successful compensated signal can be output to the host computer for further processing via UART (Universal Asynchronous Receiver Transmitter) directly. All the components are low power with high performance, which can provide high precision measurements and compensation.

### 5.2. Results of the Compensating System

**Figure 20.**
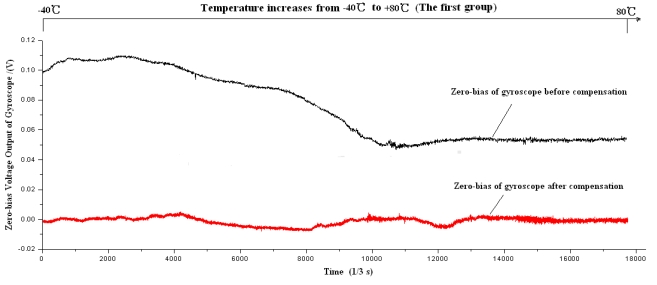
Zero bias of microgyroscope before and after compensation.

**Figure 21.**
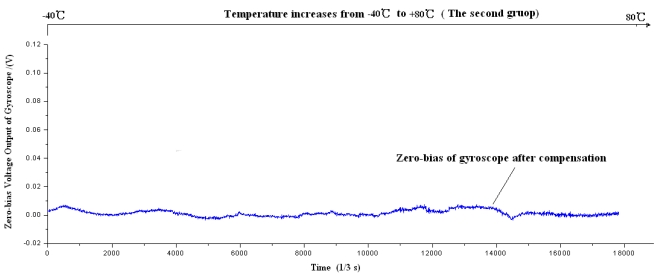
Zero bias of microgyroscope after compensation.

**Figure 22.**
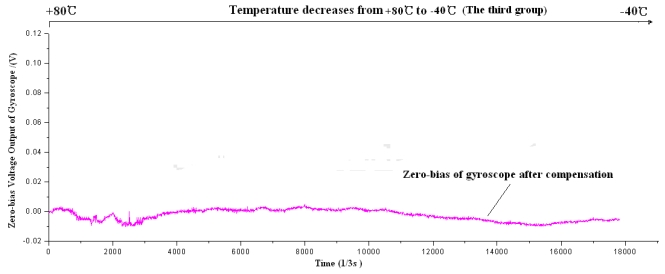
Zero bias of microgyroscope after compensation.

Firstly the piecewise polynomial fitting equations are memorized in the microprocessor. Secondly the zero bias *V_bias_* for each temperature is calculated through the formula using the tested temperature *T* as the input. Finally the actual output of gyroscope is subtracted from *V_bias_* to get the compensated zero bias shown in Figures 20 to 22.

**Figure 23.**
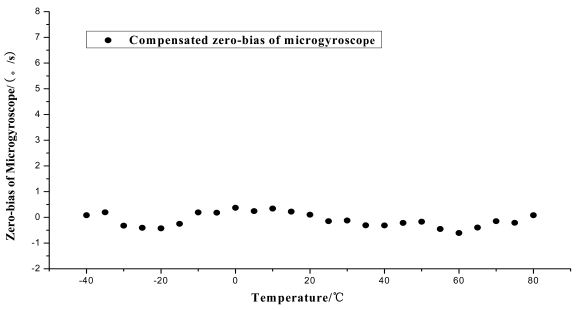
Compensated zero bias of the microgyroscope.

Firstly the uncompensated zero bias is recorded while the ambient temperature rises up from −40 °C to +80 °C. Secondly, the polynomial fitting is accomplished and the programs with the fitting equations are downloaded to the microprocessor. To verify the compensating effects, the same microgyroscope is placed in the temperature control box with no rotation and several groups of temperature experiments are carried out. In the first and second group, the ambient temperature is controlled to rise from −40 °C to +80 °C. Nevertheless in the third group the temperature descends from +80 °C to −40 °C to test the effectiveness of the model in case that ambient temperature decreases, the results show that the model can be effective when the ambient temperature descends. The compensation effects of each group are shown in Figures 20–22, respectively. The average value of the three groups of gyroscope zero bias tested is figured out with a temperature interval of 5 °C, and the processed results are shown in Table 3 and Figure 23.

As shown in Table 2 and Figure 23, the compensated zero bias of microgyroscope fluctuates mildly around zero. According to Table 2 and Table 3, the maximum absolute value of zero bias decreases from 12.3310°/s before compensation to 0.608°/s after compensation. Therefore the compensation effect of the temperature compensation system is obvious and effective.

Note: the scale factor of the tested microgyroscope is 8.774mV/(°/s).

## 6. Design of the Temperature Control System and Experimental Results

As shown in Figure 23 in Section 5, with the help of the temperature compensation, the zero bias of microgyroscope still fluctuates within a small range with the ambient temperature variation. The temperature compensation system still cannot completely compensate for the temperature effects on the performance of the microgyroscope. Moreover because of the manufacturing errors and influences of peripheral circuit devices in different microgyroscopes, their temperature models are rather different. Therefore it is desirable to find a more universally applicable method to reduce temperature effects.

**Figure 24.**
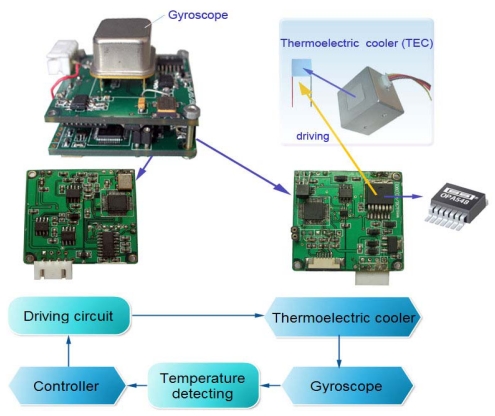
Block diagram of temperature control system.

Through abundant experiments and previous experience, it is found that the optimal working temperature for the microgyroscope is about 55 °C. It is proposed that temperature inside the packaged gyroscope should be controlled around this optimal value in order to achieve the best performance of the microgyroscope. Therefore a temperature controlling system is proposed and its effects are analyzed in detail. The temperature control system adopts a single closed-loop method. The current temperature measured by a DS18B20 temperature sensor is sent to the microprocessor. Next the tested value is compared with the set temperature value to attain the deviation value. Then the PID adjuster calculates the control value, which is subsequently transformed by the driving circuit and applied on the thermoelectric cooler (a semiconductor chip). Finally the temperature inside the gyroscope cavity is controlled around the set value. The block diagram of temperature control system is shown in Figure 24.

### 6.1. System Design

A high-current integrated driving chip OPA548 (Burr-Brown Company) is employed for driving a TEC based on the Peltier effect. Its bipolar power supply mode is used to further amplify the power for the D/A converter AD5060, implementing power driving of the thermoelectric cooler (TEC) chip.

#### 1. Temperature control algorithms

The Integral-separated PID algorithm is employed for the temperature control to ensure the integral action and decrease the overshoot. Hence the controlling performance can be improved remarkably [18-20]. The sampled-data control of the microprocessor is only capable of calculating the control-value based on the sampling time, therefore the differential coefficient and integral function need dispersing processing before application. Hence the incremental PID control algorithm is utilized in the temperature control system.

To realize an efficient control, a threshold value *ξ* > 0 is set beforehand, *e*(*k*) = *c*(*k*) − *r*(*k*) denotes the deviation between the actual temperature output *c*(*k*) and the set temperature value *r*(*k*). When |*e*(*k*)| > *ξ*, PD (Proportion Differential) control mode is operated to avoid excessive overshoot and simultaneously ensure a fast response of this system. Otherwise, when |*e*(*k*)| ≤ *ξ*, PID control mode is operated to reach the system's control precision. In other words, the basic theory of integral-separated PID algorithms is that the integral part starts working only when the casing temperature error is within the set error-band.

In the math expression, the integral part is multiplied by *β*, which is set to 1 in case of |*e*(*k*)| ≤ *ξ*. Thus the control value can be expressed as:

(17) u(k)=Ae(k)+u(k−1)−Be(k−1)+Ce(k−2

where *A* = *K_p_*(1 + *T*/*T_I_* + *T_D_*/*T*), *B* = *K_P_*(1 + 2*T_D_*/*T*), *C* = *K_P_T_D_*/*T*, and *K_p_* is the proportional coefficient.

When |*e*(*k*)| > *ξ*, *β* is set to 0, then the control value can be rewritten as:

(18) $$u(k)=A^{\prime }e(k)-B^{\prime }e(k-1)$$

where *A′* = *K_p_*(1 + *T*/*T_I_* + *T_D_*/*T*), *B′* = *K_P_*(1 + 2*T_D_*/*T*).

#### 2. Software design

Once the control system starts operation, the microprocessor will first send out the order to read the temperature. Next, the current temperature of the gyroscope T1 measured by the DS18B20 is transmitted to be compared with the set value T. Their difference is transferred in time to the PID controller. Then the control quantity calculated by the PID controller is then transformed to an analog voltage signal, which is subsequently amplified by the high current driving circuit to enhance its driving capability. Ultimately it is applied to the TEC to realize the heating or cooling function.

**Figure 25.**
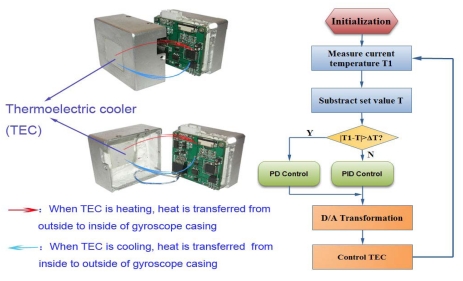
Gyroscope casing design and system software flow chart.

In order to improve the control speed, integral-separated PID control was adopted in the program development. Namely a threshold value ΔT is set beforehand. When the difference between the current temperature tested in the gyroscope is higher than or equal to ΔT (0.5 °C), the PD control method will activate, otherwise the PID control mode is used instead. Figure 25 shows the designed gyroscope casing with the mounted TEC chip and software flow chart of the control system.

Due to the lag effect of the complex thermal inertial properties and inaccurate transfer function of the inner housing of the controlled gyroscope, through lots of experimental trial, the optimal control parameters of the controller module can be decided. In this case, when the proportional coefficient *K_p_* approaches 0.48, the integral coefficient *K_I_*=*K_p_T*/*T_I_* approaches 0.32, the differential coefficient *K_D_* = *K_p_T_D_*/*T* approaches 10, and the sampling interval time is set 2 s, the closed-loop temperature control system can performance well. If the temperature in gyroscope casing is lower than the given temperature of 55 °C, the TEC starts to heat up by a positive applied voltage, and heat is transferred from the external environment to the inner housing based on the Peltier effect, while if the temperature is higher than 55 °C, the TEC starts to refrigerate by a negative applied voltage, and cooling is then transferred from the inner housing to the external environment based on the same effect.

### 6.2. Effects of Temperature Control System

**Figure 26.**
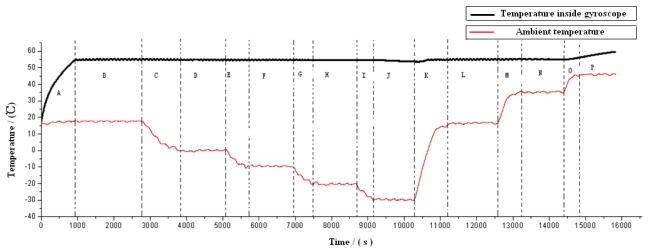
Results of controlled temperature inside the integrated microgyroscope over ambient temperature changes. A---When ambient temperature remains at normal temperature of 16 °C, temperature control system starts working. B---When ambient temperature remains at 16 °C, the temperature is recorded inside the gyroscope after it reaches 55 °C in 30 minutes. C---When ambient temperature decrease from 16 °C to 0 °C, the temperature is recorded inside the gyroscope. D---When ambient temperature is kept at 0 °C for 20 minutes, the temperature is recorded inside the gyroscope. E---When ambient temperature decrease from 0 °C to −10 °C, the temperature is recorded inside the gyroscope. F---When ambient temperature is kept at −10 °C for 20 minutes, the temperature is recorded inside the gyroscope. G---When ambient temperature decrease from −10 °C to −20 °C, the temperature is recorded inside the gyroscope. H---When ambient temperature is kept at −20 °C for 20 minutes, the temperature is recorded inside the gyroscope. I---When ambient temperature decrease from -20 °C to −30 °C, the temperature is recorded inside the gyroscope. J---When ambient temperature is kept at −30 °C for 20 minutes, the temperature is recorded inside the gyroscope. K---When ambient temperature increases from −30 °C to 16 °C, the temperature is recorded inside the gyroscope. L---When ambient temperature is kept at 16 °C for 20 minutes, the temperature is recorded inside the gyroscope. M---When ambient temperature increase from 16 °C to 35 °C, the temperature is recorded inside the gyroscope. N---When ambient temperature is kept at 35 °C for 20 minutes, the temperature is recorded inside the gyroscope. O---When ambient temperature increase from 35 °C to 45 °C, the temperature is recorded inside the gyroscope. P---When ambient temperature is kept at 45 °C for 20 minutes, the temperature is recorded inside the gyroscope.

To validate the effects of the temperature control system, the control circuit is integrated with the microgyroscope B34 investigated previously. The thermoelectric cooler (TEC) is wall-imbedded in the outside shell of the gyroscope. During the experiments, the ambient temperature is controlled by temperature control box, which descends from room temperature (16 °C) to −30 °C before rising up to 45 °C. The actual temperature inside the gyroscope and the environmental temperature are recorded and shown in Figure 26.

As shown in Figure 26, it takes approximately 900 seconds to reach the stable temperature state, and when the ambient temperature varies within −20 °C to +35 °C (Section B to Section I and Section M to Section N), the control system can effectively stabilize the maximum absolute temperature error inside the integrated microgyroscope within 0.3 °C.

**Figure 27.**
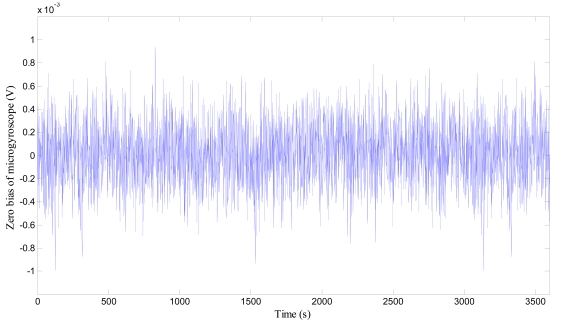
Zero bias of Microgyrosope.

**Figure 28.**
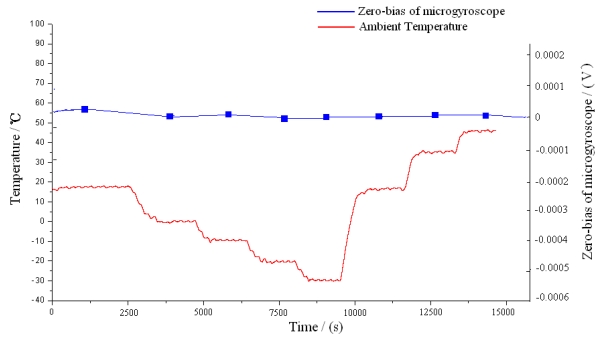
Mean zero bias of the microgyrosope under temperature compensation-control.

Because the start-up procedure needs some time and there exists constant zero bias even at the set temperature of 55 °C, the combination of the controlling and compensation methods should be jointly employed to improve the overall performance in full temperature range. Under the temperature controlling regime, the polynomial fitting compensation program is also imbedded into the C8051F360, so the compensation-control methods are working under time-sharing mode to produce nearly null zero bias in the microgyroscope. To test the temperature compensation-control effects of the system, the gyroscope prototype is placed still in the temperature control box, the ambient temperature is controlled to descend from the room temperature 16 °C to −30 °C before rising up to 45 °C. The zero bias of microgyroscope at each temperature point is recorded for sixty minutes. Figure 27 exhibits one of sixteen groups of compensated zero bias such as section D. Then the average output of the microgyroscope at each temperature point is accurately calculated, so the overall trend of zero bias over temperature changes can be seen in Figure 28.

## 7. Conclusions

The performance of a microgyroscope is greatly affected by temperature variations. In this paper the temperature testing results uncover the temperature characteristics of a microgyrocope, and validate the theoretical analysis. To improve the performance of the microgyroscope, two methods are simulated and carried out for comparison, and the experiment results demonstrate that polynominal fitting can meet the performance requirement when its term order is high enough.

In the actual developed miniaturized prototype, due to the simplicity and real-time advantage, the polynomial fitting is adopted to build the zero bias temperature model of the microgyroscope. The microprocessor compensates the actual output with the estimated value calculated by the model. The experimental results show the effectiveness of the temperature compensation system, which reduces the maximum zero bias from 12.3310°/s before compensation to 0.608°/s after compensation, which has the same order of magnitude obtained with the previous BP neural network.

In order to get the ideal working state of microgyroscope, a temperature control system is proposed to stabilize the temperature inside the casing of microgyroscope. Experimental results show that the temperature control method can effectively stabilize the temperature around 55 °C inside the integrated microgyroscope while the ambient is within −20 °C ∼ +35 °C, and the maximum stable-state error can be smaller than 0.3 °C. However, to resolve the start-up time issue, a combination of the temperature compensation and controlling methods is used to ensure the overall performance over the full temperature range. By this effective way the gyroscope and its peripheral circuits are not subject to the ambient temperature fluctuations, so the entire output of the microgyroscope can be kept at a relatively stable state. Final experimental results validate the effectiveness of the temperature compensation-control methods of the microgyroscope.

## Figures and Tables

**Figure 10.**
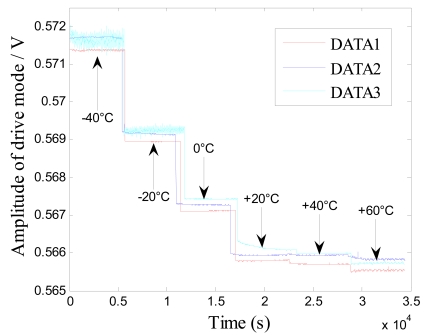
Closed-loop test of drive amplitude of microgyroscope with temperature changes.

**Figure 12.**
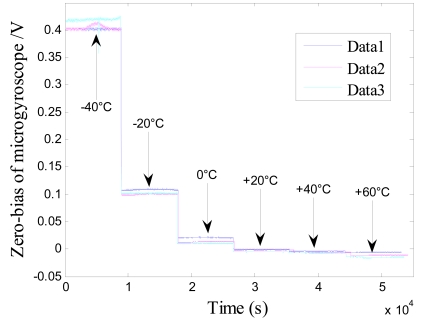
Closed-loop test of zero bias of microscope with temperature changes.

**Table 1.** Testing results of resonant frequency and quality factor.

**Temperature (°C)**	**Drive Mode**		**Sense Mode**	
	**Resonant frequency(Hz)**	**Quality factor**	**Resonant frequency(Hz)**	**Quality factor**

−40	**2,475.35**	**19,041**	**2,426.30**	**16,175**
−30	**2,474.86**	**17,678**	**2,426.20**	**15,164**
−20	**2,474.35**	**16,495**	**2,426.10**	**14,271**
−10	**2,473.50**	**15,459**	**2,425.65**	**13,476**
0	**2,473.02**	**13,739**	**2,425.16**	**12,126**
10	**2,472.30**	**10,749**	**2,424.85**	**11,022**
20	**2,471.60**	**9,886**	**2,424.30**	**9,697**
30	**2,470.20**	**8,822**	**2,423.80**	**8,097**
40	**2,469.40**	**8,232**	**2,424.10**	**6,925**
50	**2,468.90**	**7,482**	**2,423.70**	**6,215**
60	**2,468.30**	**7,052**	**2,422.90**	**6,057**

**Table 2.** Uncompensated zero bias of microgyroscope over temperature.

**Temperature (°C)**	**Average voltage output(mV)**	**Zero bias (°/s)**	**Temperature (°C)**	**Average voltage output(mV)**	**Zero bias (°/s)**
80	52.7	6.01	15	88.3	10.06
75	52.2	5.95	10	94.2	10.74
70	53.2	6.06	5	98.4	11.21
65	53.5	6.10	0	102.7	11.71
60	52.4	5.97	−5	103.5	11.80
55	50.3	5.73	−10	105.7	12.05
50	51.6	5.88	−15	108.9	12.41
45	54.5	6.21	−20	109.1	12.43
40	60.6	6.91	−25	108.2	12.33
35	68.4	7.79	−30	107.5	12.25
30	74.4	8.48	−35	105.9	12.07
25	79.9	9.11	−40	98.1	11.18
20	85.8	9.77			

Note: the scale factor of the tested microgyroscope is 8.774mV/(°/s).

**Table 3.** Results of the compensated zero bias of microgyroscope.

**Temperature (°C)**	**Average voltage output(mV)**	**Zero bias (°/s)**	**Temperature (°C)**	**Average voltage output(mV)**	**Zero bias (°/s)**
80	0.746	0.085	15	1.948	0.222
75	−1.843	−0.210	10	2.983	0.340
70	−1.316	−0.150	5	2.124	0.242
65	−2.983	−0.399	0	3.246	0.369
60	−5.334	−0.608	−5	1.597	0.182
55	−3.965	−0.452	−10	1.667	0.190
50	−1.483	−0.169	−15	−2.194	−0.250
45	−1.930	−0.219	−20	−3.772	−0.429
40	−2.773	−0.316	−25	−3.581	−0.408
35	−2.719	−0.309	−30	−2.844	−0.324
30	−1.071	−0.122	−35	1.703	0.194
25	−1.299	−0.148	−40	0.721	0.082
20	0.895	0.102			
